# Evaluation of small dense low‐density lipoprotein concentration for predicting the risk of acute coronary syndrome in Chinese population

**DOI:** 10.1002/jcla.23085

**Published:** 2019-11-07

**Authors:** Bin Wu, Zhiwu Yu, Tong Tong, Xinxin Tong, Yinmei Yang, Yongcai Tang, Huiming Ren, Yike Liao, Jun Liao

**Affiliations:** ^1^ Department of Laboratory Medicine Guangzhou First People's Hospital The Second Affiliated Hospital of South China University of Technology Guangzhou China; ^2^ Division of Laboratory Science Affiliated Cancer Hospital & Institute of Guangzhou Medical University Guangzhou China; ^3^ Department of Pharmacy Anhui Provincial Hospital The First Affiliated Hospital of University of Science and Technology of China Anhui China; ^4^ Department of Blood Transfusion Guangzhou First People's Hospital The Second Affiliated Hospital of South China University of Technology Guangzhou China; ^5^ Department of Laboratory Medicine Wuxi Fourth People's Hospital Affiliated Hospital of Jiangnan University Wuxi China; ^6^ Peking University Health Science Centre Beijing China

**Keywords:** acute coronary syndrome, Chinese population, risk factor, small dense LDL

## Abstract

**Background:**

Acute coronary syndrome (ACS) is the leading cause of death in developing and developed countries, yet assessing the risk of its development remains challenging. Several lines of evidence indicate that small, dense low‐density lipoproteins (sd‐LDL) are associated with increased cardiovascular disease risk. We aim to evaluate sd‐LDL concentration for predicting the risk of ACS in Chinese population.

**Methods:**

Baseline characteristics of 121 patients with ACS and 172 healthy controls were obtained. Plasma sd‐LDL‐C was measured using homogeneous assay, and the proportion of sd‐LDL‐C in LDL‐C was detected.

**Results:**

There was gender and age effect on the sd‐LDL‐C concentration and sd‐LDL‐C/LDL‐C ratio among healthy subjects. Elevated sd‐LDL‐C concentrations and sd‐LDL‐C/LDL‐C ratio were observed in ACS patients with unstable angina pectoris (UAP), non–ST‐segment elevation myocardial infarction (STEMI), and ST‐segment elevation myocardial infarction (NSTEMI) compared with healthy controls (*P* < .05); however, there were no differences among ACS groups. According to Pearson's correlation coefficient analyses, sd‐LDL‐C concentration and sd‐LDL‐C/LDL‐C ratio were positively correlated with triglyceride (TG) and LDL‐C concentrations (*P* < .05) and negatively correlated with high‐density lipoprotein (HDL) concentration (*P* < .05). Based on the receiver operating characteristic (ROC) curves, the cutoff values of sd‐LDL‐C and sd‐LDL‐C/LDL‐C ratio for the prediction of ACS were 1.06 mmol/L and 34.55%, respectively. Multivariate logistic regression analysis demonstrated that the sd‐LDL‐C/LDL‐C ratio, but not sd‐LDL‐C concentration, was significantly associated with ACS events [OR (95% CI): 1.24, 1.11‐1.38, *P* < .001].

**Conclusions:**

The sd‐LDL‐C/LDL‐C ratio may be associated with an increased risk of developing ACS in Chinese population.

**Abbreviations**
ACS: acute coronary syndrome
AMI: acute myocardial infarction
AUC: area under the curve
CAD: coronary artery disease
CI: confidence intervals
CK‐MB: creatine kinase MB fraction
FBG: fasting blood glucose
HDL‐C: high‐density lipoprotein cholesterol
hs‐cTnI: high‐sensitive cardiac troponin I
LDL: low‐density lipoprotein
MI: myocardial infarction
MYO: myoglobin
NMR: nuclear magnetic resonance
NSTEMI: non–ST‐segment elevation myocardial infarction
OR: odds ratio
ROC: receiver operating characteristic
sd‐LDL: small dense LDL
STEMI: ST‐segment elevation myocardial infarction
TG: triglyceride
UAP: unstable angina pectoris

## 1 INTRODUCTION

Acute coronary syndrome (ACS) is a more severe type of coronary artery disease (CAD), which contributed to a major cause of mortality and morbidity in developed and developing countries. This syndrome consists of unstable angina pectoris (UAP), non–ST‐segment elevation myocardial infarction (NSTEMI), and ST‐segment elevation myocardial infarction (STEMI).^1^ There is accumulating evidence that ACS is related to recent activation of the immune‐mediated inflammatory process associated with atherosclerotic plaque*s*.^2^ Low‐density lipoprotein (LDL) is the principal cholesterol‐carrying lipoprotein in human plasma and, as such, contributes significantly to the morbidity and mortality of CAD.^3^ Therefore, European Society of Cardiology (ESC) guidelines for preventing ACS recommend decreasing low‐density lipoprotein cholesterol (LDL‐C) to a target level of <1.8 mmol/L.^4^ Large prospective cohort studies comparing LDL particle number with LDL‐C level have demonstrated that the LDL‐C particle number was more strongly associated with the risk of CAD than the LDL‐C level.^5^, ^6^ Therefore, evaluation of LDL levels cannot identify all individuals with incident CAD, as many CAD events occur in subjects with normal LDL levels.^7^

Low‐density lipoprotein is composed of heterogeneous particles that differ in density, size, and chemical composition. LDL particles are divided into 2 distinct phenotypes: pattern A, with a higher proportion of large, more buoyant LDL particles (lb‐LDL), and pattern B, with a predominance of small dense LDL (sd‐LDL) particles.^8^ sd‐LDL particles are considered to be highly atherogenic as a result of higher penetration into the arterial wall, lower binding affinity for the LDL receptor, prolonged plasma half‐life, and lower resistance to oxidative stress compared with buoyant LDL.^9^ Using LDL‐C to evaluate cholesterol‐related CAD risk will underestimate actual risk in individuals who have optimal LDL‐C levels but high levels of sd‐LDL‐C.^10^ Due to the atherogenic properties of sd‐LDL, its measurement may be useful for risk assessment.

Small dense low‐density lipoprotein has been found to be associated with increased risk of cardiovascular disease in cross‐sectional studies and prospective observational studies using traditional measurement methods.^11^, ^12^, ^13^, ^14^, ^15^ However, to date, few studies have explored the role of sd‐LDL‐C in the diagnosis and treatment of ACS. Fukushima et al^16^ found that the sd‐LDL‐C concentration was significantly higher in patients with ACS compared with controls, especially those with metabolic syndrome, in addition, the reduction in sd‐LDL‐C by atorvastatin was much more greater than LDL‐C in patients with ACS, which indicated that sd‐LDL‐C is a superior therapeutic marker of statin treatment in patients with ACS. In general, Chinese patients typically have lower baseline levels of LDL‐C than the patients of western countries.^17^ Because of the complication of pathophysiological processes of atherothrombosis and racial differences, whether sd‐LDL‐C concentration or the sd‐LDL‐C/LDL‐C ratio is a better predictor of ACS risk than LDL‐C concentration or other traditional cardiovascular risk factors in Chinese population remains unknown.

## 2 MATERIALS AND METHODS

### 2.1 Study population

This study enrolled one hundred and seventy‐two healthy subjects who were randomly selected from consecutive subjects visiting the Guangzhou First People's Hospital for an annual health check‐up from March 2016 to February 2017. The inclusion criteria of healthy participants were as follows: (a) All participants were native Chinese aged 22‐76 years old; and (b) All participants did not have a history of CAD, thromboembolic disease, peripheral arterial disease, malignancy, infectious disease, liver or renal diseases, endocrine diseases, dyslipidemia, or diabetes mellitus. All healthy subjects were divided into groups according to age (20‐29, 30‐39, 40‐49, 50‐59, and ≥60 years old) and sex (male and female).

Patients with ACS who satisfied all criteria for inclusion were selected. The patients with ACS enrolled in the present study had UAP (31 men and 17 women), NSTEMI (23 men and six women), and STEMI (37 men and seven women). ACS was diagnosed according to criteria established by the European Society of Cardiology^18^, ^19^: Briefly, acute myocardial infarction (MI) was defined as a typical rise, which was defined as >99% of normal levels [troponin T > 0.05 ng/mL; creatine kinase MB fraction (CK‐MB) >10 ng/mL], and gradual fall of troponin, or a more rapid rise and fall of CK‐MB, with at least one of the following: acute onset of typical ischemic chest pain; some Q waves in V1‐V3, 30‐ms Q waves ≥1 mm in two contiguous leads; ST‐segment elevation or depression in ≥2 leads, ≥0.2 mV in V1‐V3, >0.1 mV in other leads. UAP was defined as a history of new‐onset, more frequent, more persistent or resting episodes of chest pain without typical changes in myocardial enzymes but with ECG evidence of myocardial ischemia (transient ST‐segment displacement >0.1 mV during chest pain).

This study was carried out in accordance with the principles of the Declaration of Helsinki and was approved by the ethics committees of Guangzhou First People's Hospital.

### 2.2 Baseline examination

Fasting blood samples were obtained by venipuncture after the participants had fasted for at least 10 hours and centrifuged immediately. The diagnosis of hypertension was based on a history of hypertension, systolic blood pressure above 140 mm Hg, or diastolic blood pressure above 90 mm Hg. The average of the second and third measurements of blood pressure was used for analysis. Diabetes mellitus was defined as a fasting glucose level ≥7.0 mmol/L (126 mg/dL) or a self‐reported history of physician‐diagnosed diabetes or treatment for diabetes. Information on smoking status and other medical histories of the patients were obtained through surveys by a well‐trained nurse.

### 2.3 Laboratory measurements

Plasma fasting blood glucose (FBG), triglyceride (TG), high‐density lipoprotein cholesterol (HDL‐C), and LDL cholesterol (LDL‐C) concentrations were measured by standard laboratory procedures in an automatic biochemistry analyzer (AU5800; Beckman Coulter) using detection kits (Beckman Coulter). In addition, plasma CK‐MB, myoglobin (MYO), and high‐sensitive cardiac troponin I (hs‐cTnI) were measured by a UniCel DxI 800 analyzer (Beckman Coulter) with commercial detection kits (Beckman Coulter).

For this study, we used archived plasma samples of healthy controls and patients with ACS that had been frozen at −80°C and never previously thawed. The determination of sd‐LDL‐C concentrations was performed by a fully automated homogeneous method (Denka Seiken Co., Ltd.) in an automated biochemistry analyzer (AU5800; Beckman Coulter) as previously described.^20^

The ratio of sd‐LDL‐C/LDL‐C was calculated as measured sd‐LDL‐C (mmol/L)/ LDL‐C (mmol/L).^18^ All the laboratory measurements of patients with ACS and healthy subjects were under no treatment after admission.

### 2.4 Statistical analysis

Statistical analyses were conducted with SPSSII for Windows (19.0). Continuous data are presented as the mean ± SD and compared with Student's *t* test. Correlation analyses for non‐parametric (Spearman's Rho) data were performed to establish relationships between sd‐LDL‐C concentration or the sd‐LDL‐C/LDL‐C ratio and traditional cardiovascular risk factors. A one‐way analysis of variance (ANOVA) and Fisher's protected least significant difference were used to compare the mean values of sd‐LDL‐C and the sd‐LDL‐C/LDL‐C ratio among groups. Receiver operating characteristic (ROC) curve analysis was used to determine the ability of sd‐LDL‐C concentration, sd‐LDL‐C/LDL‐C ratio, and LDL‐C concentration to predict ACS events. To examine the association of sd‐LDL‐C and the sd‐LDL‐C/LDL‐C ratio with ACS events, we performed a multivariate logistic regression analysis; all odds ratios (ORs) are presented with their 95% confidence intervals (CIs). *P* < .05 was considered statistically significant.

## 3 RESULTS

### 3.1 Baseline characteristics

Baseline characteristics of all patients with ACS and healthy subjects at admission to the hospital are provided in Table 1. The mean ages of the patients in the UAP, STEMI, and NSTEMI groups were 65.70, 64.44, and 63.93 years, respectively. All the study participants were Chinese, and over 60% was male.

**Table 1:** Baseline characteristics of ACS patients and healthy subjects

Characteristics	Healthy subjects	ACS patients			
		UAP	STEMI	NSTEMI	*P* value
Patients—no. (%)	172	48 (39.7)	44 (36.3)	29 (24.0)	
Age—y	42.3 ± 16.7	65.70 ± 1.60	64.44 ± 2.10	63.93 ± 2.18	NS
Male sex—no. (%)	77 (44.8)	33 (68.8)	33 (75.0)	23 (79.3)	NS
Risk factors					
Hypertension	/	33 (68.8)	22 (50.0)	17 (58.6)	0.048
Diabetes mellitus	/	14 (29.2)	13 (29.5)	9 (31.0)	NS
History of smoking	/	16 (33.3)	17 (38.6)	12 (41.4)	NS
Laboratory values on admission					
Myoglobin—ng/mL	54.7 ± 2.9	134.7 ± 3.6	144.8 ± 28.6	138.1 ± 19.1	0.037
Creatine kinase MB—ng/mL	1.9 ± 0.2	2.4 ± 0.3	19.6 ± 7.6	11.0 ± 2.4	0.015
Creatinine—μmol/L	75.4 ± 4.3	95.3 ± 5.3	105.1 ± 14.6	111.1 ± 20.9	NS
Hs‐cTnI—ng/mL	0.031 ± 0.012	0.048 ± 0.020	1.982 ± 0.877	1.184 ± 0.378	0.003
Triglyceride—mmol/L	1.2 ± 0.1	2.0 ± 0.4	1.5 ± 0.1	1.9 ± 0.3	NS
HDL‐C—mmol/L	1.1 ± 0.1	1.2 ± 0.1	1.1 ± 0.1	1.1 ± 0.1	NS
LDL‐C—mmol/L	1.7 ± 0.1	2.4 ± 0.1	3.0 ± 0.1	2.9 ± 0.2	NS

Note

Values are mean ± SD or percent.

Abbreviation: NS, Not Significant.

### 3.2 sd‐LDL‐C concentrations and sd‐LDL‐C/LDL‐C ratios in healthy subjects

The precision and accuracy of the automated homogeneous assay were evaluated by analyzing 20 replicates of quantitation standard samples before this study. The precision (CV %) of this method was 2.1%, and the diagnostic accuracy was 96.9% (data not shown), which showed similar diagnostic performance as previous study.^19^

**Figure 1:**
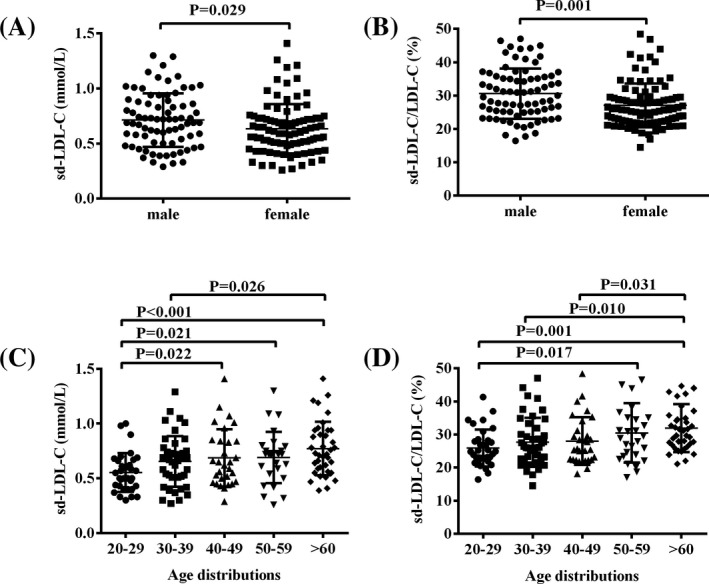
The concentration of sd‐LDL‐C and the sd‐LDL‐C/LDL‐C ratio in healthy subject. A, B, Gender difference between healthy males and females at the concentration of sd‐LDL‐C and the sd‐LDL‐C/LDL‐C ratio. C, D, Age difference between healthy males and females at the concentration of sd‐LDL‐C and the sd‐LDL‐C/LDL‐C ratio

One hundred and seventy‐two subjects were classified into the male and female groups. The age of the subjects ranged from 22 to 76 years old, and the mean age was 42.3 ± 16.7 years. To determine whether there was an age or a gender difference, we first compared the sd‐LDL‐C concentration and sd‐LDL‐C/LDL‐C ratio between females and males (Figure 1A,B) and then separated the healthy female and male participants into five age groups (Figure 1C,D). The sd‐LDL‐C concentration was significantly higher in males than in females (0.71 ± 0.24 vs 0.63 ± 0.22 mmol/L, *P* = .029); similarly, the sd‐LDL‐C/LDL‐C ratio was significantly higher in males than in females (30.61 ± 7.51 vs 27.08 ± 6.59%, *P* = .001). In addition, the sd‐LDL‐C concentration was significantly higher in the age groups of 40‐49, 50‐59, and above 60 years than in the age group of 20‐29 years (0.68 ± 0.26, 0.69 ± 0.23, and 0.77 ± 0.25 vs 0.55 ± 0.18 mmol/L; *P* = .022, .021, and <.001, respectively). The sd‐LDL‐C/LDL‐C ratio in the age groups of 50‐59 and above 60 years was significantly higher than that in the 20‐29‐year age group (30.48 ± 9.01 and 31.89 ± 7.27 vs 25.95 ± 5.58%; *P* = .017 and .001, respectively). Additionally, the sd‐LDL‐C/LDL‐C ratio in the above‐60‐years age group was significantly higher than that in the 30‐ to 39‐ and 40‐ to 49‐year age groups (27.64 ± 7.40 and 28.00 ± 7.20 vs 30.48 ± 9.01%; *P* = .010 and .031, respectively).

### 3.3 Association of sd‐LDL‐C and the sd‐LDL‐C/LDL‐C ratio with cardiovascular risk factors

The correlations of the sd‐LDL‐C concentration and sd‐LDL‐C/LDL‐C ratio with various traditional cardiovascular risk factors are shown in Table 2. Strong positive correlations of sd‐LDL‐C concentration and the sd‐LDL‐C/LDL‐C ratio with lipid risk factors, such as log triglycerides (*P* < .001 and *P* < .001, respectively) and LDL‐C (*P* = .003 and *P* < .001, respectively), were observed. In addition, HDL‐C concentration showed a negative correlation with sd‐LDL‐C concentration and the sd‐LDL‐C/LDL‐C ratio (*P* = .013 and *P* < .001, respectively). Additionally, hs‐cTnI was not significantly correlated with sd‐LDL‐C concentration and the sd‐LDL‐C/LDL‐C ratio.

**Table 2:** Correlation of sd‐LDL‐C and sd‐LDL‐C/LDL‐C ratio with traditional cardiovascular risk factors

Risk factors	sd‐ LDL‐C		sd‐LDL‐C/LDL‐C	
	Pearson R	*P*	Pearson *R*	*P*
HDL‐C	−0.223	.013	−0.325	<.001
LDL‐C	0.597	<.001	0.265	.003
hs‐cTnI	0.164	.089	0.237	.013
Log triglycerides	0.356	<.001	0.528	<.001

### 3.4 sd‐LDL‐C concentrations and ACS events

**Figure 2:**
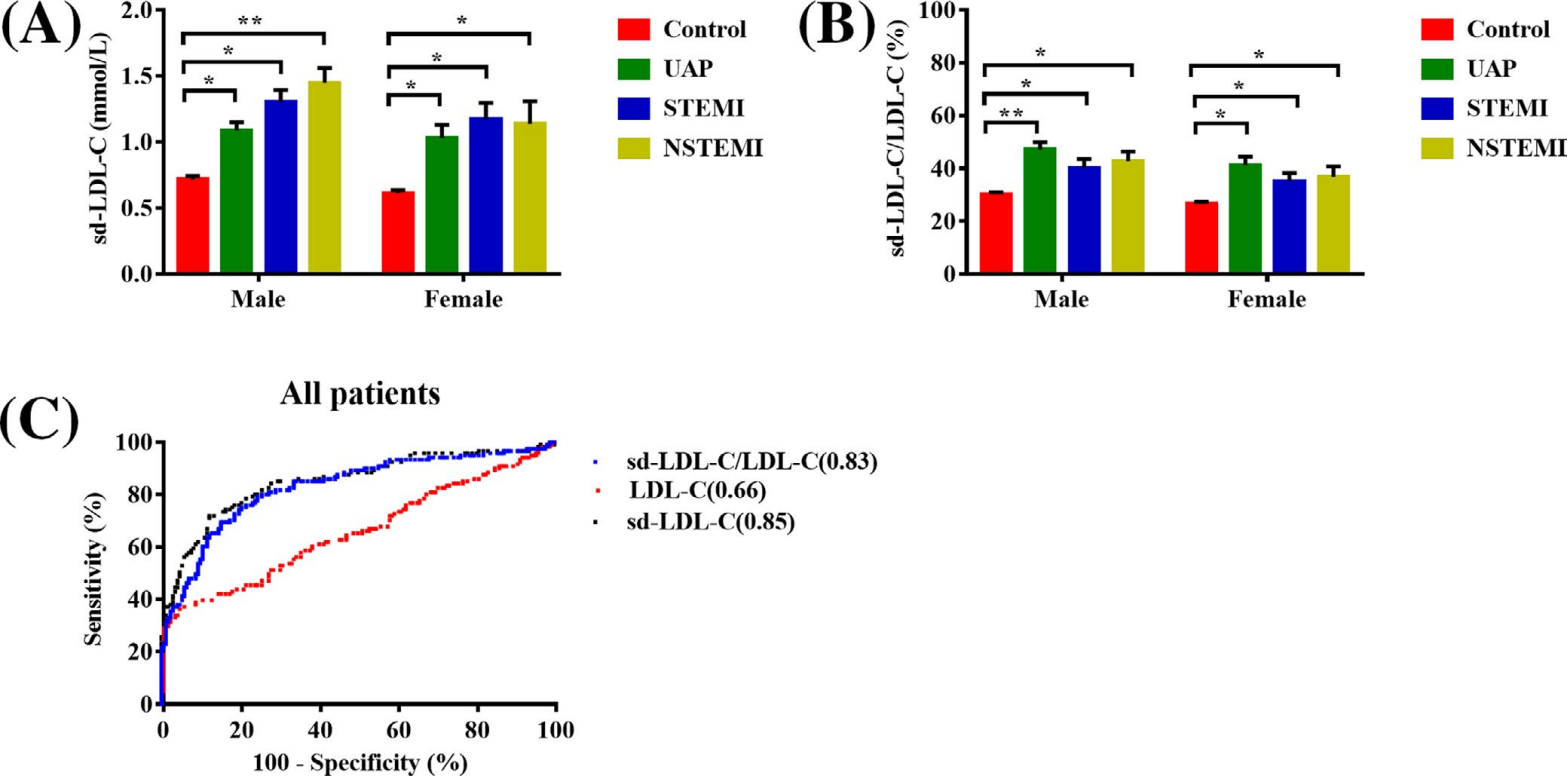
The concentration of sd‐LDL‐C and the sd‐LDL‐C/LDL‐C ratio and ROC curves of ACS patients. A, B, The concentration of sd‐LDL‐C and the sd‐LDL‐C/LDL‐C ratio in patients with UAP, STEMI and NSTEMI and healthy controls in males and females. C, ROC curves of sd‐LDL‐C concentration, the sd‐LDL‐C/LDL‐C ratio, and LDL‐C level in ACS patients

To eliminate the effects of gender and age, we selected the group of above 50 years old of healthy subjects as normal control and divided them into male and female groups. Among all the male participants, the mean baseline plasma sd‐LDL‐C concentration was 0.72 (healthy control), 1.09 (UAP), 1.30 (STEMI), and 1.44 (NSTEMI) mmol/L, and the mean sd‐LDL‐C/LDL‐C ratio was 30.27% (healthy control), 47.26% (UAP), 40.04% (STEMI), and 42.81% (NSTEMI). Among all the female participants, the mean baseline plasma sd‐LDL‐C concentration was 0.62 (healthy control), 1.03 (UAP), 1.17 (STEMI), and 1.14 (NSTEMI) mmol/L, and the mean sd‐LDL‐C/LDL‐C ratio was 26.71%(healthy control), 41.41% (UAP), 35.25% (STEMI), and 36.87% (NSTEMI) (Figure 2A,B). sd‐LDL‐C concentrations and sd‐LDL‐C/LDL‐C ratios were significantly higher in ACS patients with UAP, STEMI, and NSTEMI than in healthy controls. In this study, we aimed to determine a cutoff value for sd‐LDL‐C concentration and the sd‐LDL‐C/LDL‐C ratio that could predict ACS. ROC analysis showed that the area under the curve (AUC) of the sd‐LDL‐C/LDL‐C ratio was highest, demonstrating that the sd‐LDL‐C/LDL‐C ratio was a more valuable predictor of ACS: [AUC (95% CI): sd‐LDL‐C/LDL‐C ratio, 0.89, 0.84‐0.93, *P* < .001; sd‐LDL‐C concentration, 0.85, 0.80‐0.90, *P* < .001; LDL‐C, 0.66, 0.60‐0.72, *P* < .001] (Figure 2C). However, there was no significant difference in the AUC between the sd‐LDL‐C concentration and sd‐LDL‐C/LDL‐C ratio. Based on the ROC curve, the cutoff values of sd‐LDL‐C and the sd‐LDL‐C/LDL‐C ratio that could predict ACS were 1.06 mmol/L and 34.55%, respectively, with a sensitivity of 71.9% and 75.2%, respectively, and a specificity of 87.8% and 92.0%, respectively (Figure 2C).

To evaluate the influence of sd‐LDL‐C concentrations and sd‐LDL‐C/LDL‐C ratios on the occurrence of ACS events, we performed a logistic regression analysis. In the univariate model, a high sd‐LDL‐C/LDL‐C ratio was significantly associated with the risk of ACS [OR (95% CI): 1.24, 1.11‐1.38, *P* < .001], while high sd‐LDL‐C concentrations were not significantly associated with an increased risk of ACS events [OR (95% CI): 0.09, 0.004‐1.94, *P* > .05]. In the multivariate model, after adjustments for age and gender (model 1), the association between the sd‐LDL‐C/LDL‐C ratio and ACS events remained significant [OR (95% CI): 1.24, 1.15‐1.35, *P* < .001]. When HDL‐C was excluded from the multivariate model (model 2), age, LDL‐C concentration, and the sd‐LDL‐C/LDL‐C ratio were significantly associated with ACS events [OR (95% CI): 1.09, 1.04‐1.13, *P* < .001; 6.97, 2.23‐21.78, *P* < .05; and 1.29, 1.17‐1.43, *P* < .01, respectively]. Similarly, when LDL‐C was excluded from the multivariate model (Model 3), age, HDL‐C concentration, and the sd‐LDL‐C/LDL‐C ratio were significantly associated with ACS events [OR (95% CI): 1.09, 1.04‐1.14, *P* < .01; 0.004, 0.000‐0.05, *P* < .01; and 1.22, 1.10‐1.34, *P* < .001, respectively] (Table 3).

**Table 3:** Predictors of ACS events according to logistic regression analysis

Variable	Univariate model		Multivariate model					
	OR	95% CI	Model 1		Model 2		Model 3	
			OR	95% CI	OR	95% CI	OR	95% CI
Age	1.08	1.03‐1.14**	1.08	1.04‐1.12***	1.09	1.04‐1.13***	1.09	1.04‐1.14**
men	2.47	0.53‐10.38	3.71	1.29‐10.72*	3.37	0.99‐11.42	2.39	0.62‐9.25
sd‐LDL‐C	0.09	0.004‐1.94	0.59	0.20‐1.79	0.04	0.003‐0.58*	0.47	0.09‐2.58
LDL‐C	3.79	0.95‐15.14	**–**	**–**	6.97	2.23‐21.78**	**–**	**–**
sd‐LDL‐C/LDL‐C	1.24	1.11‐1.38***	1.24	1.15‐1.35***	1.29	1.17‐1.43**	1.22	1.10‐1.34***
HDL‐C	0.01	0.001‐0.17**	**–**	**–**	**–**	**–**	0.004	0.000‐0.05**

Note

Model 1 was adjusted for age, men, sd‐LDL‐C, and sd‐LDL‐C/LDL‐C.

Model 2 was adjusted for age, men, sd‐LDL‐C, sd‐LDL‐C/LDL‐C, and LDL‐C.

Model 3 was adjusted for age, men, sd‐LDL‐C, sd‐LDL‐C/LDL‐C, and HDL‐C.

* *P* < .05.

** *P* < .01.

*** *P* < .001.

## 4 DISCUSSIONS

In the present study, we investigated the relationship between plasma concentrations of sd‐LDL‐C and risk of ACS using a newly developed automated homogeneous sd‐LDL‐C assay. Our results showed that elevated concentrations of sd‐LDL‐C and elevated sd‐LDL‐C/LDL‐C ratios were strongly associated with ACS.

Small dense low‐density lipoprotein is traditionally measured by ultracentrifugation^21^ or gradient gel electrophoresis,^22^ which separates different LDL particles by density and size; however, both methods are laborious and time‐consuming and require special equipment. Nuclear magnetic resonance (NMR) imaging can be used to simultaneously determine the size and number of LDL particles, but the instrument required for NMR is too expensive; therefore, NMR is not yet widely available for routine clinical practice, although automated NMR analyzers have already been developed for high‐throughput clinical laboratory testing.^23^ Heparin sodium salt precipitation followed by centrifugation enables the determination of sd‐LDL concentrations; however, this method still requires offline sample pre‐treatment, which hinders its integration into general clinical practice.^24^ Due to the above limitations, the traditional methodologies for the assessment of sd‐LDL are not suitable for use in clinical laboratories in large‐scale samples. Ito et al^20^ developed a fully automated homogeneous method for detection of sd‐LDL, which showed good agreement with traditional method and high specificity. Then, this method was widely used in understanding the significance of sd‐LDL in the following clinical studies.^25^ Though the homogeneous sd‐LDL–automated homogeneous assay is available already for almost 8 years, however, this precise and rapid method for the routine measurement of large numbers of samples was introduced into clinical laboratory of China within 3 years.

We used this method to explore the diagnosis performance of patients with ACS of Chinese population and found that there are gender and age differences in sd‐LDL‐C concentrations and sd‐LDL‐C/LDL‐C ratios among healthy controls, which is consistent with the data obtained in Western and Chinese populations.^26^, ^27^, ^28^ As demonstrated in the previous studies, pattern B was reported in approximately 25% of the population but was less frequent in women and younger subjects (40 years old).^29^ In addition, CAD risk markedly increases with age in men and after menopause in women, and alterations in LDL concentration clearly contribute to this increased risk.^30^ However, the present study did not separate menopausal from premenopausal women to explore the effects of menopause on sd‐LDL‐C.

Few studies have demonstrated the clinical value of sd‐LDL‐C in the assessment of ACS risk. Kwon et al^31^ found that sd‐LDL‐C was an independent risk factor for the development of ACS in the Korean population by using polyacrylamide tube gel electrophoresis method. In addition, Emadzadeh et al^32^ demonstrated that sd‐LDL‐C was significantly higher in patients presenting with ACS than controls and showed no differences between acute myocardial infarction (AMI) and UAP groups. The automated homogeneous sd‐LDL‐C assay we used in this study can directly detect the plasma concentration of sd‐LDL‐C in large numbers of samples within a short time. And we also found that the mean sd‐LDL‐C concentration and sd‐LDL‐C/LDL‐C ratio were significantly higher in the ACS group than in the healthy control group in Chinese population. However, increases in sd‐LDL‐C concentration did not show a statistically significant trend toward an increased risk of CAD (OR, 1.008), whereas increases in the sd‐LDL‐C/LDL‐C ratio showed a 5.78‐fold increased risk for CAD.^33^ Similarly, we demonstrated that a high sd‐LDL‐C/LDL‐C ratio was associated with a 1.25‐fold higher risk of ACS (*P* < .001), while sd‐LDL‐C concentration was not significantly associated with ACS (*P* > .05). Therefore, a high sd‐LDL‐C/LDL‐C ratio might be a more important risk factor for cardiovascular events among patients with ACS than a high sd‐LDL‐C concentration.

Several previous studies have assessed the associations of sd‐LDL‐C with traditional cardiovascular risk factors.^3^ In agreement with previous studies, we also found that plasma sd‐LDL‐C concentrations and the sd‐LDL‐C/LDL‐C ratio were positively correlated with plasma TG and LDL‐C concentrations; however, sd‐LDL‐C concentrations and sd‐LDL‐C/LDL‐C ratios were negatively correlated with HDL‐C concentration. Thus, these data suggest that sd‐LDL‐C is adversely associated with cardiovascular lipid risk factors and that increased concentrations of sd‐LDL‐C and high sd‐LDL‐C/LDL‐C ratios may be associated with metabolic disorders.

Our data suggest that the sd‐LDL‐C/LDL‐C ratio is a promising novel parameter for the assessment of ACS risk. The advantage of the automated homogeneous assay for determining sd‐LCL‐C content is excellent reproducibility, which makes this assay much more user‐friendly and more applicable than traditional methods. However, the present study also some limitations: First, the cohort was relatively small; therefore, additional large cohort studies are required to clarify the predictive value of the sd‐LDL‐C/LDL‐C ratio, as measured by the homogeneous assay, in subjects with ACS. Second, the ACS study population represented a heterogeneous cohort with regards to gender. Third, the plasma we used in our study was previously stored at −80°C; therefore, whether the results of this study can be extended to fresh plasma samples remains to be determined.

## References

[1] Kim HK , Jeong MH , Ahn Y , et al. A new risk score system for the assessment of clinical outcomes in patients with non‐ST‐segment elevation myocardial infarction. Int J Cardiol. 2010;145(3):450‐454. 19541376 10.1016/j.ijcard.2009.06.001

[2] Ehara S , Ueda M , Naruko T , et al. Elevated levels of oxidized low density lipoprotein show a positive relationship with the severity of acute coronary syndromes. Circulation. 2001;103(15):1955‐1960. 11306523 10.1161/01.cir.103.15.1955

[3] Hoogeveen RC , Gaubatz JW , Sun W , et al. Small dense low‐density lipoprotein‐cholesterol concentrations predict risk for coronary heart disease: the Atherosclerosis Risk In Communities (ARIC) study. Arterioscler Thromb Vasc Biol. 2014;34(5):1069‐1077. 24558110 10.1161/ATVBAHA.114.303284 PMC3999643

[4] Gencer B , Koskinas KC , Raber L , et al. Eligibility for PCSK9 Inhibitors According to American College of Cardiology (ACC) and European Society of Cardiology/European Atherosclerosis Society (ESC/EAS) Guidelines After Acute Coronary Syndromes. J Am Heart Assoc. 2017;6(11):e006537. 29122809 10.1161/JAHA.117.006537 PMC5721754

[5] Otvos JD , Mora S , Shalaurova I , Greenland P , Mackey RH , Goff DC Jr . Clinical implications of discordance between low‐density lipoprotein cholesterol and particle number. J Clin Lipidol. 2011;5(2):105‐113. 21392724 10.1016/j.jacl.2011.02.001 PMC3070150

[6] El Harchaoui K , van der Steeg WA , Stroes ES , et al. Value of low‐density lipoprotein particle number and size as predictors of coronary artery disease in apparently healthy men and women: the EPIC‐Norfolk Prospective Population Study. J Am Coll Cardiol. 2007;49(5):547‐553. 17276177 10.1016/j.jacc.2006.09.043

[7] Rana JS , Liu JY , Moffet HH , et al. Metabolic dyslipidemia and risk of coronary heart disease in 28,318 adults with diabetes mellitus and low‐density lipoprotein cholesterol <100 mg/dl. Am J Cardiol. 2015;116(11):1700‐1704. 26428026 10.1016/j.amjcard.2015.08.039

[8] Zhao X , Sun D , Xu RX , et al. Low‐density lipoprotein‐associated variables and the severity of coronary artery disease: an untreated Chinese cohort study. Biomarkers. 2018;23(7):647‐653. 29730953 10.1080/1354750X.2018.1474256

[9] Kaneva AM , Potolitsyna NN , Bojko ER . Usefulness of the LDL‐C/apoB ratio in the overall evaluation of atherogenicity of lipid profile. Arch Physiol Biochem. 2017;123(1):16‐22. 27347637 10.1080/13813455.2016.1195411

[10] Tsai MY , Steffen BT , Guan W , et al. New automated assay of small dense low‐density lipoprotein cholesterol identifies risk of coronary heart disease: the Multi‐ethnic Study of Atherosclerosis. Arterioscler Thromb Vasc Biol. 2014;34(1):196‐201. 24233487 10.1161/ATVBAHA.113.302401 PMC4211254

[11] Austin MA , Breslow JL , Hennekens CH , Buring JE , Willett WC , Krauss RM . Low‐density lipoprotein subclass patterns and risk of myocardial infarction. JAMA. 1988;260(13):1917‐1921. 3418853

[12] Campos H , Genest JJ Jr , Blijlevens E , et al. Low density lipoprotein particle size and coronary artery disease. Arterioscler Thromb. 1992;12(2):187‐195. 1543692 10.1161/01.atv.12.2.187

[13] Gardner CD , Fortmann SP , Krauss RM . Association of small low‐density lipoprotein particles with the incidence of coronary artery disease in men and women. JAMA. 1996;276(11):875‐881. 8782636

[14] Stampfer MJ , Krauss RM , Ma J , et al. A prospective study of triglyceride level, low‐density lipoprotein particle diameter, and risk of myocardial infarction. JAMA. 1996;276(11):882‐888. 8782637

[15] Lamarche B , Tchernof A , Moorjani S , et al. dense low‐density lipoprotein particles as a predictor of the risk of ischemic heart disease in men. Prospective results from the Quebec Cardiovascular Study. Circulation. 1997;95(1):69‐75. 8994419 10.1161/01.cir.95.1.69

[16] Fukushima Y , Hirayama S , Ueno T , et al. Small dense LDL cholesterol is a robust therapeutic marker of statin treatment in patients with acute coronary syndrome and metabolic syndrome. Clin Chim Acta. 2011;412(15–16):1423‐1427. 21530500 10.1016/j.cca.2011.04.021

[17] Tomlinson B , Chan P , Liu ZM . Statin responses in chinese patients. J Atheroscler Thromb. 2018;25(2):199‐202. 28740057 10.5551/jat.40204 PMC5827089

[18] Satoh N , Wada H , Ono K , et al. Small dense LDL‐cholesterol relative to LDL‐cholesterol is a strong independent determinant of hypoadiponectinemia in metabolic syndrome. Circ J. 2008;72(6):932‐939. 18503219 10.1253/circj.72.932

[19] Albers JJ , Slee A , Fleg JL , O'Brien KD , Marcovina SM . Relationship of baseline HDL subclasses, small dense LDL and LDL triglyceride to cardiovascular events in the AIM‐HIGH clinical trial. Atherosclerosis. 2016;251:454‐459. 27320173 10.1016/j.atherosclerosis.2016.06.019 PMC4983241

[20] Ito Y , Fujimura M , Ohta M , Hirano T . Development of a homogeneous assay for measurement of small dense LDL cholesterol. Clin Chem. 2011;57(1):57‐65. 21051530 10.1373/clinchem.2010.149559

[21] Hirano T , Ito Y , Yoshino G . Measurement of small dense low‐density lipoprotein particles. J Atheroscler Thromb. 2005;12(2):67‐72.15942115

[22] Austin MA , King MC , Vranizan KM , Krauss RM . Atherogenic lipoprotein phenotype. A proposed genetic marker for coronary heart disease risk. Circulation. 1990;82(2):495‐506. 2372896 10.1161/01.cir.82.2.495

[23] Matyus SP , Braun PJ , Wolak‐Dinsmore J , et al. NMR measurement of LDL particle number using the Vantera Clinical Analyzer. Clin Biochem. 2014;47(16–17):203‐210. 25079243 10.1016/j.clinbiochem.2014.07.015

[24] Hirano T , Ito Y , Koba S , et al. Clinical significance of small dense low‐density lipoprotein cholesterol levels determined by the simple precipitation method. Arterioscler Thromb Vasc Biol. 2004;24(3):558‐563. 14726414 10.1161/01.ATV.0000117179.92263.08

[25] Hirayama S , Miida T . Small dense LDL: an emerging risk factor for cardiovascular disease. Clin Chim Acta. 2012;414:215‐224. 22989852 10.1016/j.cca.2012.09.010

[26] Ai M , Otokozawa S , Asztalos BF , et al. Small dense LDL cholesterol and coronary heart disease: results from the Framingham Offspring Study. Clin Chem. 2010;56(6):967‐976. 20431054 10.1373/clinchem.2009.137489 PMC3791882

[27] Shen H , Xu L , Lu JF , et al. Correlation between small dense low‐density lipoprotein cholesterol and carotid artery intima‐media thickness in a healthy Chinese population. Lipids Health Dis. 2015;14:137. 26510458 10.1186/s12944-015-0143-x PMC4625741

[28] Shen H , Zhou J , Shen GR , Yang HL , Lu ZY , Wang HM . Correlation between serum levels of small, dense low‐density lipoprotein cholesterol and carotid stenosis in cerebral infarction patients >65 years of age. Ann Vasc Surg. 2014;28(2):375‐380. 24200130 10.1016/j.avsg.2013.01.029

[29] Beltrame JF , Sasayama S , Maseri A . Racial heterogeneity in coronary artery vasomotor reactivity: differences between Japanese and Caucasian patients. J Am Coll Cardiol. 1999;33(6):1442‐1452. 10334407 10.1016/s0735-1097(99)00073-x

[30] Expert Panel on Detection, Evaluation, and Treatment of High Blood Cholesterol in Adults Executive summary of the third report of the National Cholesterol Education Program (NCEP) expert panel on detection, evaluation, and treatment of high blood cholesterol in adults (adult treatment panel III). JAMA. 2001;285(19):2486‐2497. 11368702 10.1001/jama.285.19.2486

[31] Kwon SW , Yoon SJ , Kang TS , et al. Significance of small dense low‐density lipoprotein as a risk factor for coronary artery disease and acute coronary syndrome. Yonsei Med J. 2006;47(3):405‐414. 16807992 10.3349/ymj.2006.47.3.405 PMC2688162

[32] Emadzadeh MR , Alavi MS , Soukhtanloo M , et al. Changes in small dense low‐density lipoprotein levels following acute coronary syndrome. Angiology. 2013;64(3):216‐222. 22539803 10.1177/0003319712441855

[33] Nishikura T , Koba S , Yokota Y , et al. Elevated small dense low‐density lipoprotein cholesterol as a predictor for future cardiovascular events in patients with stable coronary artery disease. J Atheroscler Thromb. 2014;21(8):755‐767. 24717762 10.5551/jat.23465

